# 

**Published:** 1895-11-09

**Authors:** 


### CONTENTS.

**Some Practical Aspects of Bovine Tuberculosis** - - 91
**Electricity and the Processes of Life** - - - - - - 92
**On Modern Progress in Ophthalmic Medicine and Surgery**, By R. B. Carter, F.R.C.S.—Squint (*continued*) - 93
**Modern Sociology.**—A Social Reform Club - - - - - 94
**Notes and News** - - - - - - - - - - - - 96
**Medical Progress and Hospital Clinics—**
The Treatment of Abscess in Pott's Disease (*contiuned*) - - 97
Progress in Medicine.—Graves' Disease (*continued*)—Diseases of the Digestive Organs - - - - - - - - - - 99
Progress in Obstetrics - - - - - - - - - - - 101
**Annotations.**—Medical Men and Medical Women—A Gleam of Hope for the Cancer-stricken—The Parents' Point of View - 95
**Editor's Letter-box** - - - - - - - - - - - 102
**The Institutional Workshop—**
Hospital Expenditure.—The Commissariat. V.—The Proportion of Servants - - - - - - - - - 103
Hospital Construction—Cottage Hospital, Wood Green - - 104
Metropolitan Hospital Sunday Fund - - - - - - 105
Hospital Festivals, Meetings, &c. - - - - - - 106
Practical Points - - - - - - - - - - - 106
**Scraps and Gleanings** - - - - - - - - - - 106

### EXTRA SUPPLEMENT.—THE NURSING MIRROR.

**News from the Nursing World.**—Visitors at Westminster Hospital—Princess Beatrice at Edinburgh—The Trained Nurses' Club—Nurses at Worcester Infirmary—Nurses as Stallholders—Young Women's Christian Association—Nottingham Children's Hospital—Prepaid Replies—The Colney Hatch Flyleaf—West Rainton District Nurse—Benwell Nurse Society—Night Nurses at Sligo—Fire at a Children's Hospital—Notes from Sydney, N.S.W.—Nursing at Bellevue, U.S.A.—Short Items - - - - - - - xxxix
**On Urine Testing.**—II.—Albumen and Specific Gravity - xliii
**Royal British Nurses' Association** - - - - - xlii
**Elementary Physiology for Nurses.** By C. F. Marshall, late Surgical Registrar, Hospital for Sick Children, Great Ormond Street. XIV.—The Nervous System (*concluded*)—The Sense Organs - - - - - - - - xli
**Where to Go** - - - - - - - - - - - xlii
**Royal National Pension Fund for Nurses** - - - xliii
**Notes and Queries** - - - - - - - - - - xliii
**Nursing in India** - - - - - - - - - - xliv
**Appointments** - - - - - - - - - - - xliv
**Dress and Uniforms** - - - - - - - - - xlv
**Everybody's Opinion** - - - - - - - - - xlvi

